# Postural control and contingent negative variation during transient floor translation while standing with the ankle fixed

**DOI:** 10.1186/s40101-016-0104-8

**Published:** 2016-07-25

**Authors:** Vitalii Lytnev, Katsuo Fujiwara, Naoe Kiyota, Mariko Irei, Hiroshi Toyama, Chie Yaguchi

**Affiliations:** 1 Department of Orthopaedic Surgery, Graduate School of Medical Science, Kanazawa University, 13-1 Takara-machi, Kanazawa, 920-8641 Japan; 2 Department of Sports and Health, Kanazawa Gakuin University, 10 Sue-machi, Kanazawa, 920-1392 Japan; 3 Department of Rehabilitation, Japan Health Care College, 6-17-3 Megumino-nishi, Eniwa, 061-1373 Japan; 4 Department of Rehabilitation Science, Osaka Health Science University, 1-9-27 Tenma, Kita-ku, Osaka 530-0043 Japan; 5 Department of Physical Therapy, Hokkaido Bunkyo University, 5-196-1 Kogane-chuo, Eniwa, 061-1449 Japan

**Keywords:** Floor translation, Contingent negative variation, Postural control, Joint fixation

## Abstract

**Background:**

Adaptation changes in postural muscle activity and anticipatory attention were investigated with the ankle joint fixed to change postural control strategies during transient floor translation.

**Methods:**

For 15 healthy young adults, 40 transient floor translations (S2) in the anterior direction were applied 2 s after an auditory warning signal (S1), under conditions with or without fixation of the ankle. Activity of the frontal postural muscles (tibialis anterior (TA), rectus femoris (RF), rectus abdominis) and contingent negative variation (CNV, brain potential) were analyzed for 20 trials each of the early and latter halves under each fixation condition.

**Results:**

With fixation, peak amplitude of muscle activity after S2 was significantly decreased in TA and increased in RF. These muscles showed marked adaptive decreases. The early component of CNV reduced with adaptation, particularly under fixation condition. Only in RF, background activity increased just before S2, with adaptation under fixation. A significant correlation was found between timings of CNV peak and RF activation just before S2 only after adaptation under fixation.

**Conclusion:**

These results suggest that the main activation muscle changes from TA to RF with fixation. Under such condition, attention would be focused on the knee with adaptation, and the need for heightening attention in the early stage may have declined. Correspondingly, the timing to heighten stiffness of the RF became later, and attention would have been paid to RF activation just before S2.

## Background

The standing posture is a human characteristic; however, its control dynamics is highly complicated. Many researchers have investigated postural responses to external perturbations while standing to clarify the dynamic characteristics of the postural control system [1–4]. Transient forward floor translation can be applied to cause backward disturbance [5, 6]. The main joint for postural control with floor translation has been reported as the ankle [3]. However, many degrees of freedom in joints and muscles are available to facilitate the maintenance of upright standing [7]. Postural control strategies in response to external disturbances could thus vary between both individuals and situations [3, 8]. If the main joint (ankle) for postural control cannot be used due to reasons such as aging or impairment, postural control strategies would change considerably [9–12]. However, few studies have investigated changes in postural control strategy during backward disturbance or the process of adaptation to a new strategy in detail [13, 14].

Joint fixation, which decreases the degrees of freedom, is one method for changing the postural control strategy. Fujiwara et al. [15] fixed all joints of the leg and trunk above the ankle during transient floor translation. Although postural control was focused on the triceps surae, fixation of joints other than the ankle could not change the postural control strategy largely because the ankle was mainly used also in postural control to the transient floor translation without the fixation. On the other hand, fixation of the ankle will allow us to investigate changes in the postural control strategy and adaptation processes. During transient forward floor translation, the tibialis anterior (TA) activates mainly against the backward postural disturbance, pivoting at the ankle [3, 16]. If the ankle joint were fixed, backward disturbance would occur around the knee joint and the main activation muscle would thus change to the rectus femoris (RF). With repetition of postural disturbances during floor oscillation, as a comparatively novel task, activation of postural muscles would adaptively decrease [17]. Therefore, according to postural adaptation with ankle joint fixation, RF activation would decrease.

We have previously investigated the relationship between postural muscle activities and contingent negative variation (CNV), obtained by averaging the electroencephalogram (EEG) recorded between warning (S1) and imperative (S2) stimuli [15, 16, 18–21]. The late component of CNV reflects the motor preparation process and anticipatory attention directed to S2 [22, 23]. Late CNV shows a peak just before S2, which has been suggested to correspond to a peak of anticipatory attention and/or onset of attentional shift to objects other than S2, such as sensory information and output of motor commands [20, 24]. When S1 was a simple warning and S2 was an imperative stimulus of finger response or arm movement, the negative potential of late CNV seemed to gradually increase until S2 onset [21, 25]. On the other hand, when S2 was a transient floor translation, the negative potential seemed to increase at a relatively early stage of the S1–S2 period [16, 26]. When the response to S2 was determined by S1 or trial was considerably repeated, the negative potential of early CNV was small [27, 28]. These findings suggest that when early preparation for the response to S2 is allowed and needed, CNV would increase negatively in the early period of S1–S2. In the floor translation task, subjects should direct their attention to and prepare for S2, since a rapid, large disturbance is applied just after S2. However, if the necessity for anticipatory attention and postural preparation in the early period is reduced by postural adaptation under ankle joint fixation, the negative potential in the early period of S1–S2 will be decreased.

In a floor translation task with predictable timing of the disturbance (i.e., S1–S2 paradigm), continuous or transient activities in the main postural muscles have been observed prior to S2, relating to increasing muscle stiffness against the disturbance or control of standing position to moderate the influence of disturbance, respectively [15, 16, 20]. These would represent the strategies of postural preparation for the disturbance. In forward floor translation, background activity of TA was continuously increased before S2 [16]. Therefore, when forward translation is applied with the ankle joint fixed, increased muscle stiffness would be observed in the RF. Then, if the necessity for postural preparation is decreased with postural adaptation under ankle fixation, the increase in the time to muscle stiffness is expected to be close to S2 onset. Such a change in RF activity with adaptation may correspond to the change in CNV with adaptation.

In addition, the start time of these muscle activations for postural preparation is reportedly earlier than the peak time for late CNV, with a high correlation between these times [16, 20]. After postural adaptation to the backward floor translation with fixation of the knee, hip, and trunk, a high correlation with CNV peak time was found for the start time of triceps surae activation [15]. These findings suggest that attention would be directed to the sensory information processing related to the activity in the main muscle preceding the disturbance. With the advance of postural adaptation in ankle joint fixation, attention will be directed primarily to RF, the main muscle for postural control following the disturbance, and consequently, a high correlation will be observed between CNV peak time and start time of RF activation before S2. However, the level of correlation would be lower than that with fixation of the upper part above the ankle, because of the greater degrees of freedom.

In the present study, the ankle joint was fixed to change the postural control strategy, and adaptation changes in postural muscle activation and anticipatory attention to forward floor translation were investigated. Working hypotheses were as follows. First, with ankle joint fixation, the focus of postural control would change from TA to RF, and RF activation would then decrease with postural adaptation. Second, with postural adaptation under ankle joint fixation, negative potential in the early period of S1–S2 would be decreased, and the timing of the start of background activity in RF before S2 would be delayed. Third, a high correlation would also be observed between CNV peak time and the start time of RF activity before S2.

## Methods

### Subjects

Fifteen healthy young adults (8 men, 7 women) participated in this experiment. Mean (standard deviation (SD)) age, height, weight, foot length (FL), and auditory threshold were 22.7 (4.7) years, 166.9 (8.3) cm, 60.0 (8.8) kg, 24.4 (1.4) cm, and 28.0 (4.6) dB, respectively. No subject had any history of neurological or orthopedic impairment. Informed consent was obtained from all subjects following an explanation of the experimental protocols, which were approved by the ethics committee at Kanazawa University (No. 946).

### Apparatus and data recording

A force platform (FPA34; Electro Design, Nagareyama, Japan) was used to measure the center of pressure in the anteroposterior direction (CoPap). CoPap signals were sent simultaneously to one computer (PC9801BX2; NEC, Tokyo, Japan) to determine CoPap position online and to another computer for analysis offline. The former received CoPap data via an A/D converter (PIO9045; I/O-Data, Kanazawa, Japan) at 20 Hz with 12-bit resolution and could generate a buzzer sound when CoPap was within ±1 cm of the position for the quiet standing (QS) posture. During QS, the frequency of body sway is below 5 Hz; especially, the main frequency is below 1.5 Hz [29, 30]. Therefore, the 20-Hz sampling frequency is commonly used in the studies on the postural sway [31]. CoPap position was calculated as the percentage distance from the heel in relation to FL (%FL). The platform was fixed to a handmade table that was movable horizontally in an anteroposterior direction by a linear motion guide actuator (SKR4610A-0290-1-1001; THK, Tokyo, Japan) with a computer-controlled electric motor (SANMOTION model No. PB PBBR604; Sanyo Denki, Tokyo, Japan). The direction, velocity, and amplitude of platform movement were adjusted by the motor. S1 was an auditory stimulus delivered via earphones at a frequency of 2000 Hz, 35 dB above the threshold and lasting 50 ms. S2 was a forward floor translation. Floor translation was detected by an accelerometer (AS-2GB; Kyowa, Tokyo, Japan) fixed to the platform.

The position of the body in the sagittal plane was recorded using the Position Sensor System (C5949; Hamamatsu Photonics, Hamamatsu, Japan). This system comprises a sensor head and light-emitting diode (LED) targets and emits analog outputs of the coordinates of the LED targets in two dimensions. The sensor head was placed 4 m from the left side of the subject. LED targets were placed over the platform and the following landmarks on the left side: vertebra prominens (C7); midpoint of the greater trochanter (hip); lateral condyle of the femur (knee); and lateral malleolus (ankle). The x- and y-coordinates of LED targets were recorded at a 0.3-mm resolution.

Ag-AgCl cup electrodes (8-mm diameter) for recording EEG were placed on the scalp at Fz, Cz, and Pz in accordance with the international 10-20 system, and referenced to linked ear lobes. A ground electrode was placed at Fpz. Electrooculography (EOG) was recorded from a pair of electrodes placed above and below the right eye. To fix the eye position, subjects were instructed to gaze at a fixation point presented on an eye-trek face-mounted display (FMD011F; Olympus, Tokyo, Japan). Surface electrodes (P-00-S; Ambu, Ballerup, Denmark) were used in bipolar derivation to record surface electromyography (EMG) of the following muscles on the right side: rectus abdominis (RA), erector spinae (ES), RF, biceps femoris (BF), TA, medial head of gastrocnemius (GcM), and soleus (Sol). For each muscle, electrodes were fixed after shaving and cleaning the skin with alcohol. The electrodes were aligned along the long axis of the muscle with an inter-electrode distance of about 3 cm. Electrode input impedance was <5 kΩ. Signals from electrodes were amplified (EEG, ×40000; EOG, ×4000; EMG, ×4000) and band-pass filtered (EEG, 0.05–100 Hz; EOG, 0.05–30 Hz; EMG, 5–500 Hz) using an amplifier (Biotop 6R12; NEC-Sanei, Tokyo, Japan). In many CNV studies with 2-s inter stimulus interval, the high-pass filter around 0.05 Hz has been used [32–35].

For subsequent analyses, all electrical signals including CoPap, EEG, EOG, and EMG were sent to the computer for analysis (Dimension E521; Dell, Kawasaki, Japan) via an A/D converter (ADA16-32/2(CB)F; Contec, Osaka, Japan) at 1000 Hz with 16-bit resolution.

### Joint fixation of the ankle

**Fig. 1:**
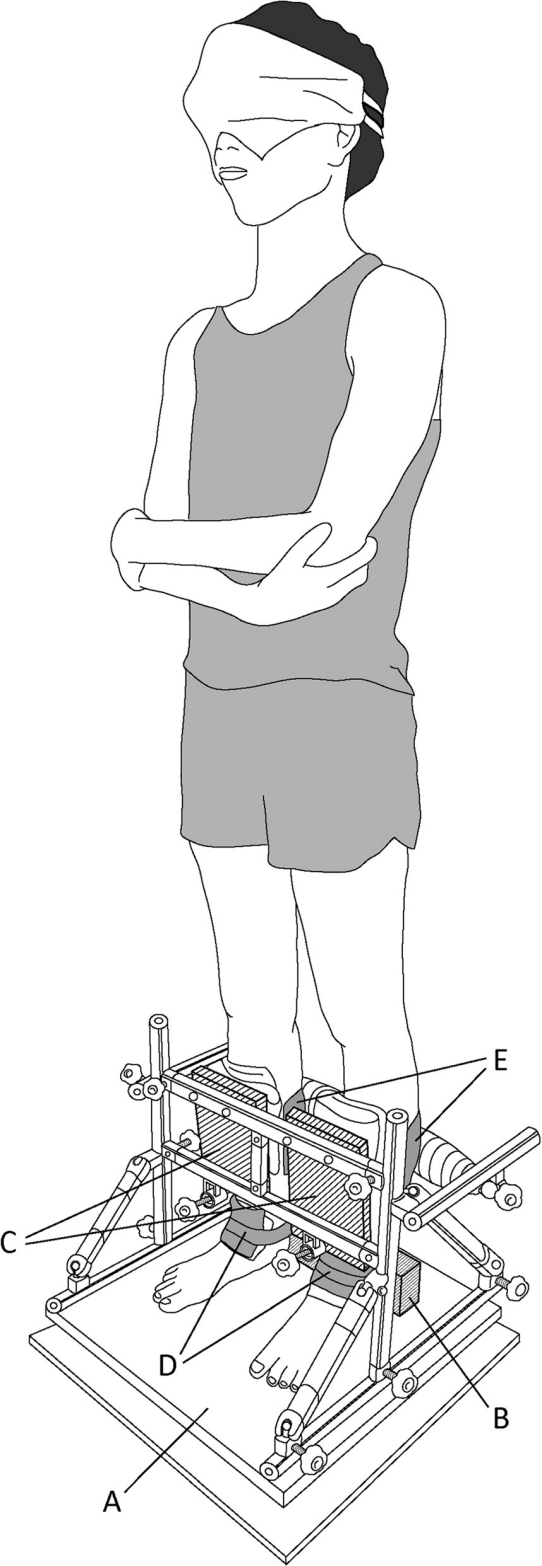
Experimental setup. (*A*) Force platform, (*B*) wooden block, (*C*) wooden board, (*D*) belt for the foot, (*E*) belt for the leg

The outline of the method for ankle joint fixation is shown in Fig. 1. The lower legs and feet were fixed using aluminum frames and a wooden block mounted on the platform. While the subject maintained QS posture with the heels slightly touching the wooden block, the legs were secured with three horizontal bars of the frames, two from the front of the legs and another from the back, and two belts each for the left and right legs. The front bars had two wooden boards along with the long axis of the legs, for which the angle of inclination could be adjusted to the legs. Fixation of foot position was ensured by wrapping the ankle joint and wooden block together with another two belts for each foot. To avoid overly tight wrapping, a towel or sponge was placed between the legs and fixation tool. During these fixation processes, the buzzer sound was continually generated to indicate QS posture.

### Procedure

All measurements were performed while the subject stood barefoot, with feet 10 cm apart and parallel on the force platform and the upper limbs crossed in front of the chest (Fig. 1). Mean CoPap position was initially measured for 10 s with the subject maintaining QS posture. The mean value of five trials was adopted as the QS position. Next, mean CoPap position during extreme backward leaning (EBL) was measured twice. Subjects gradually leaned backward from QS posture for approximately 5 s, pivoting at the ankles, and then maintained this EBL posture for 3 s. The more posterior mean CoPap position of two trials was adopted as the EBL mean position, and the posterior peak position of CoPap in the adopted trial was defined as the EBL peak position.

Velocity and amplitude of floor translation was set for each subject based on EBL mean and peak positions [16, 36]. To begin, a 5- or 10-cm floor translation was applied at a velocity of 10 cm/s. If the posterior peak of CoPap after translation at either amplitude was located between EBL mean position and EBL peak position, 10 cm/s was adopted as the translation velocity. If not, velocity was reduced or increased until the posterior peak at either amplitude was located between these positions (change in 5-cm/s increments). Second, a linear regression line was drawn through the two coordinates of the floor translation amplitude (5 and 10 cm) and the posterior peak of CoPap at each of the two floor translation amplitudes at the determined velocity. Based on this line, the translation amplitude at which the posterior peak would be located between the EBL mean position and EBL peak position was determined. Mean (SD) values for adopted translation velocity and amplitude were 20.0 (5.0) cm/s and 7.3 (2.3) cm, respectively.

The experimental session was carried out as follows. In both setting the translation intensity and in the experimental session, subjects maintained CoPap position within the QS position ±1 cm, as presented by a buzzer sound for at least 3 s, until S2 onset. S1 was randomly presented 1–2 s after the experimenter stopped the buzzer sound, then S2 started 2 s after S1. Subjects were instructed to avoid changing the initial foot position in response to S2. A set of 20 trials was repeatedly performed twice for each condition (early- and latter-half sets). Trials were excluded if a change in foot position was observed or if CoPap deviated more than ±1 cm from QS position before S2. Subjects were given a standing rest period of 30 s between trials and a seated rest period of 3 and 10 min between every ten trials and between fixation conditions, respectively.

### Data analyses

All data analyses were performed using BIMUTAS II software (Kissei Comtec, Nagano, Japan). To evaluate the magnitude of backward disturbance in response to floor translation, the posterior peak of CoPap after S2 was identified in each trial and the distance from EBL mean position to this peak position was calculated. The mean value from all trials in each set was defined as the CoPap displaced position.

EEG, EMG, CoPap, and position sensor data from 500 ms before S1 to 3000 ms after S2 were averaged for each set. Trials with eye blinks or movement artifacts (voltage at EOG or any EEG electrode exceeding ±100 μV) between 500 ms before S1 and S2 were excluded from averaging. At least 12 trials were included in the average for each set, as the minimum number of averagings in previous CNV studies [37]. For EEG, mean amplitude for the 500-ms period before S1 was used as a baseline of averaging. Prior to averaging, EMG data were high-pass filtered at 40 Hz using a seventh-order Butterworth filter to exclude electrocardiographic and movement artifacts, then full-wave rectified. For position sensor data, waveforms of the x- and y-coordinates were first smoothed by an 89-point moving average, corresponding to a 5-Hz low-pass filter. Hip angle (knee − hip − C7), knee angle (ankle − knee − hip), and ankle angle (inclination of ankle − knee from vertical line) were then calculated from the coordinates, using Excel 2010 software (Microsoft, Tokyo, Japan). Joint angles were calculated for every data point, and the waveform was then averaged.

In the averaged waveforms after S2, joint angle and EMG were analyzed. Movement angle of each joint was defined as the difference between maximal and minimal values after S2. EMG was analyzed only in the frontal postural muscles (RA, RF, and TA), since the direction of postural disturbance was backward only and burst activity just after S2 was observed mainly in these muscles. In order to smooth this averaged EMG waveforms, an 11-point moving average corresponding to a 40-Hz low-pass filter was used with reference to the previous studies [3, 38]. The maximum peak after S2 for these muscles was identified, and peak amplitude and latency were measured relative to the baseline and S2, respectively.

For analysis in the period before S2, averaged EEG, EMG, and CoPap waveforms were smoothed by a 111-point moving average, corresponding to a 4-Hz low-pass filter. CNV peaked just before S2 and then changed positively in 12 of 15 subjects, and in the other 3 subjects, the negative potential was not clearly increased. Thus, the following analyses between S1 and S2 were performed for the 12 subjects. Mean amplitudes for every 100-ms period from 100 ms before S1 to S2 onset were then calculated. CNV analyses used averaged EEG waveforms recorded from Cz, in which late CNV was maximal in all sets. CNV can be classified into early and late components [39]. The early component has been reported as the potential between 300 and 700 ms after S1, and the late component as the negative potential, which gradually increases toward S2 following the early component [40–42]. The periods from 700 ms after S1 to S2 were thus used for the analysis of mean amplitudes for every 100 ms of CNV, EMG, and CoPap. For the mean amplitude of CoPap, the difference from the mean amplitude for the 500-ms period before S1 was also calculated, since forward deviation was observed early after S1.

A maximal negative potential identified from 1400 ms after S1 to S2 was defined as the CNV peak, and the latency relative to S2 was calculated as CNV peak time. Changing patterns of frontal muscle activity and CoPap movement around the CNV peak were analyzed as follows [20]. The 500-ms period from 700 to 200 ms before the CNV peak was defined as the base period. For EMG, a continuous increase in preparation for S2 was identified as activation exceeding 2 SD above the mean amplitude of the base period for >50 ms until S2 and the minimum value of this activation was defined as the onset point. When a continuous increase of EMG was not observed, presence of a CoPap forward shift exceeding 2 SD above the mean amplitude in the base period until S2 was checked. If such a CoPap forward shift was observed, the inflection point was identified based on the second-order differentiated waveform and the start of the transient increase in EMG activity just before the inflection of the CoPap shift was identified in the same manner as the start of the continuous increase. The time difference between the start of the increase in EMG and S2 was defined as the EMG start time before S2.

### Statistical analyses

Shapiro-Wilk tests confirmed that all data satisfied assumptions of normal distribution. Two-way repeated-measures analysis of variance (ANOVA) was used to assess the main effects and interaction of condition (no-fixation, fixation) and set (early half, latter half) on analysis parameters after S2, mean amplitude of CNV, EMG, and CoPap for every 100-ms period between S1 and S2, and CNV peak time and peak amplitude. When a significant interaction was shown, paired *t* tests were used for post hoc comparison to investigate differences within each factor. For EMG peak latency after S2, to assess whether effects of condition (no-fixation, fixation) and set (early half, latter half) differed among muscles (RA, RF, TA), three-way ANOVA was performed first, then the post hoc Games-Howell test was used to further investigate differences among muscles. To investigate the effects of joint fixation, a paired *t* test was used to compare each parameter across the latter half under the no-fixation condition and the early half under the fixation condition. To investigate changing patterns of CNV and EMG waveforms in the period corresponding to the late CNV, with the mean amplitude for every 100-ms period, the difference between the period of 700–800 ms after S1 and other periods was evaluated using Dunnett’s test. A one-sample *t* test was used to assess whether the CoPap displaced position and CoPap mean amplitude for every 100-ms period between S1 and S2 differed significantly from the EBL mean position and mean amplitude for the 500-ms period before S1, respectively. Pearson’s correlation was used to assess the relationship between CNV peak time and EMG start time before S2. The alpha level was set at *p* < 0.05. All statistical analyses were performed using IBM SPSS Statistics 19 (IBM Japan, Tokyo, Japan).

## Results

The statistical results of ANOVA are shown in Table 1. The results of main effects and post hoc tests are described in the text with corresponding *p* values.

**Table 1:** Results of analysis of variance (ANOVA)

Dependent variables	Significant interaction or main factor	*F* values	Significance
Two-way ANOVA			
CoPap displaced position	Set	*F*_1,14_ = 22.0	*p* < 0.001
Hip joint movement angle	Set	*F*_1,14_ = 5.7	*p* < 0.05
Knee joint movement angle	Interaction	*F*_1,14_ = 6.5	*p* < 0.05
Ankle joint movement angle	Condition	*F*_1,14_ = 19.6	*p* < 0.01
	Set	*F*_1,14_ = 14.0	*p* < 0.01
Peak amplitude of RA after S2	Interaction	*F*_1,14_ = 5.0	*p* < 0.05
Peak amplitude of RF after S2	Condition	*F*_1,14_ = 7.2	*p* < 0.05
	Set	*F*_1,14_ = 23.0	*p* < 0.001
Peak amplitude of TA after S2	Condition	*F*_1,14_ = 118.0	*p* < 0.001
	Set	*F*_1,14_ = 103.6	*p* < 0.001
CNV peak amplitude	Nothing	*F*_1,11_ < 1.1	N.S.
CNV peak time	Condition	*F*_1,11_ = 9.8	*p* < 0.05
Mean amplitude of CNV from 700 to 800 ms after S1	Condition	*F*_1,11_ = 4.9	*p* < 0.05
Mean amplitude of CNV from 800 to 900 ms after S1	Condition	*F*_1,11_ = 5.2	*p* < 0.05
	Set	*F*_1,11_ = 5.4	*p* < 0.05
Mean amplitude of CNV from 900 to 1500 ms after S1	Set	*F*_1,11_ = 5.7	*p* < 0.05
Mean amplitude of CNV from 1600 ms after S1 to S2	Nothing	*F*_1,11_ = 2.8	N.S.
Mean amplitude of RA from 700 ms after S1 to S2	Nothing	*F*_1,11_ = 3.3	N.S.
Mean amplitude of RF from 700 ms after S1 to S2	Nothing	*F*_1,11_ = 4.6	N.S.
Mean amplitude TA from 700 to 1500 ms after S1	Nothing	*F*_1,11_ < 4.5	N.S.
Mean amplitude of TA from 1600 ms after S1 to S2	Interaction	*F*_1,11_ > 5.0	*p* < 0.05
Mean amplitude of CoPap from 700 ms after S1 to S2	Condition	*F*_1,11_ > 8.0	*p* < 0.05
Tree-way ANOVA			
Peak latency of frontal postural muscles after S2	Interaction between condition and muscle	*F*_2,42_ = 6.8	*p* < 0.01

If a significant interaction was found, the statistical value of that interaction alone was described. If significant main factors were found, but no interactions, only the statistical values of the main factors were described

**Fig. 2:**
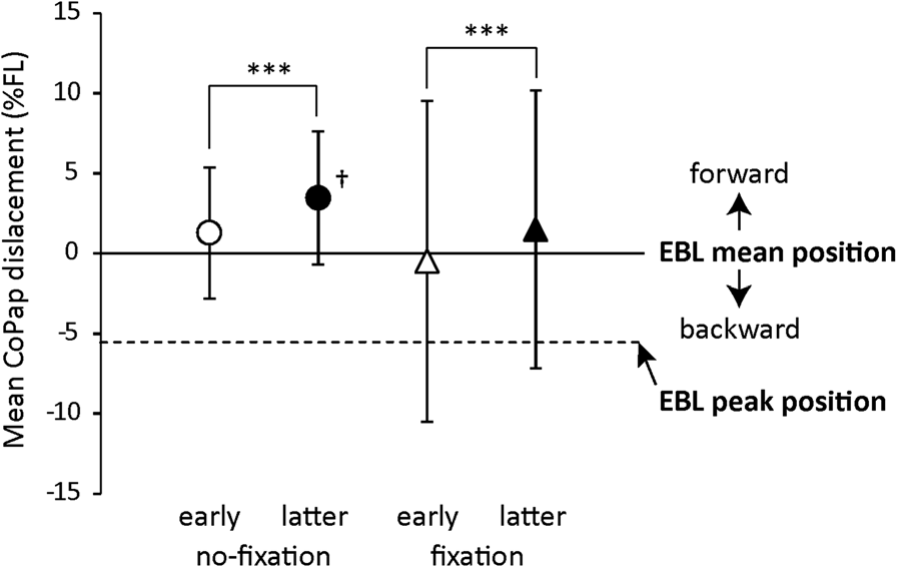
Results of CoPap displaced positions in response to floor translation. ***p < 0.001, †significantly forward of EBL mean position

Figure 2 shows mean CoPap displaced position after S2. The mean displaced position was significantly forward in the latter half compared to the early half (*p* < 0.001), regardless of fixation condition. Under the no-fixation condition, position in the latter half was significantly forward of the EBL mean position (*t*_14_ = 3.2, *p* < 0.01).

**Fig. 3:**
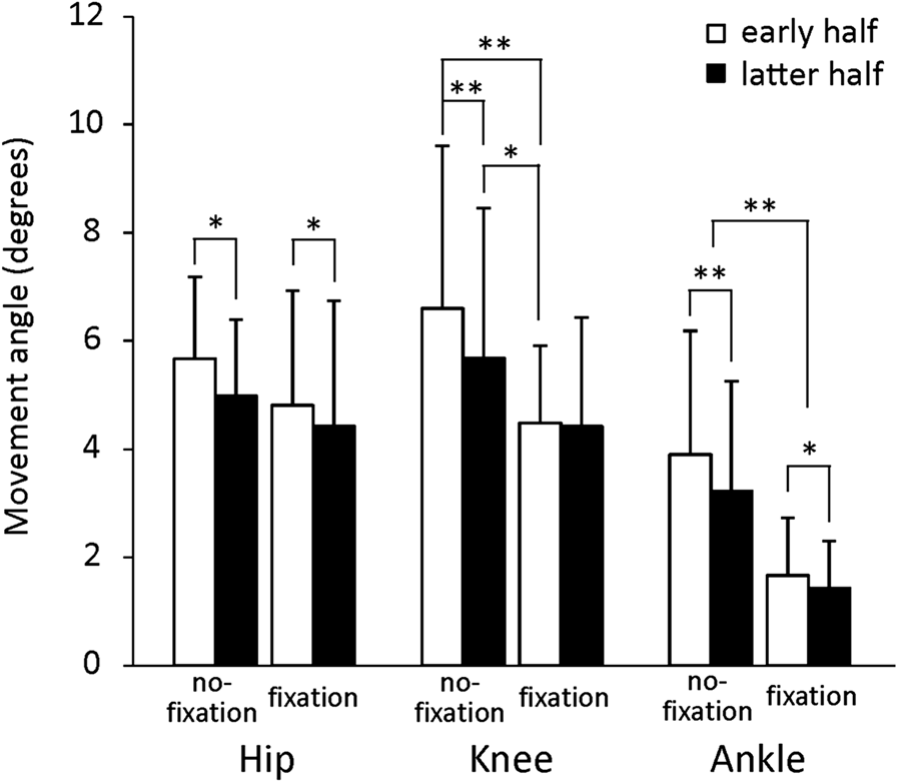
Movement angles of the hip, knee and ankle in response to floor translation. *p < 0.05, **p < 0.01

Figure 3 shows joint movement angle after S2. In the no-fixation condition, movement angle was significantly smaller in the latter half than in the early half for all three joints (*p* < 0.05). Movement angles of the ankle and knee were significantly smaller in the early half under the fixation condition than in the early and latter half under the no-fixation condition (*p* < 0.05). In the fixation condition, movement angles of the ankle and hip were significantly smaller in the latter half than in the early half (*p* < 0.05), whereas that of the knee showed no significant change.

**Fig. 4:**
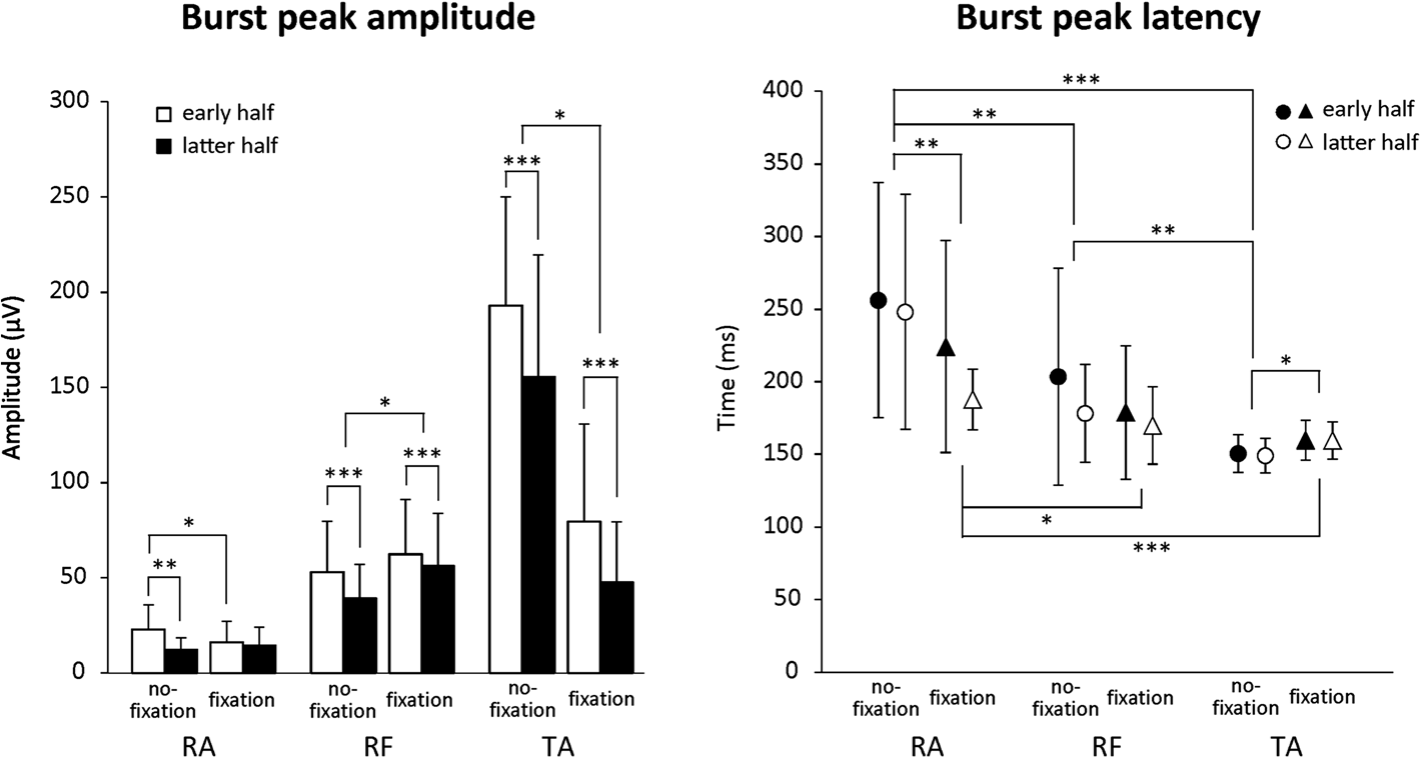
Burst peak amplitude and latency of postural muscles in response to floor translation. *p < 0.05, **p < 0.01, ***p < 0.001

Figure 4 shows burst peak amplitude and latency of postural muscle after S2. In the no-fixation condition, burst peaks occurred in order of TA, RF, and RA, with significant differences among muscles (*p* < 0.01), regardless of set. In the fixation condition, burst peak latencies of TA and RF were significantly earlier than that of RA (*p* < 0.05), and no significant difference was found between TA and RF, regardless of set. Compared to the no-fixation condition, peak latency under fixation was significantly delayed in TA (*p* < 0.05) and shortened in RA (*p* < 0.01). Peak amplitudes of all three muscles were significantly decreased in the latter half than in the early half under the no-fixation condition (*p* < 0.01). With fixation, the amplitude increased significantly in RF (*p* < 0.05) and decreased in TA (*p* < 0.05), regardless of set. In the latter half under fixation, amplitudes of TA and RF decreased significantly compared to the early half (*p* < 0.001). For RA, peak amplitude in the early half was significantly smaller in the fixation condition than in the no-fixation (*p* < 0.05).

**Fig. 5:**
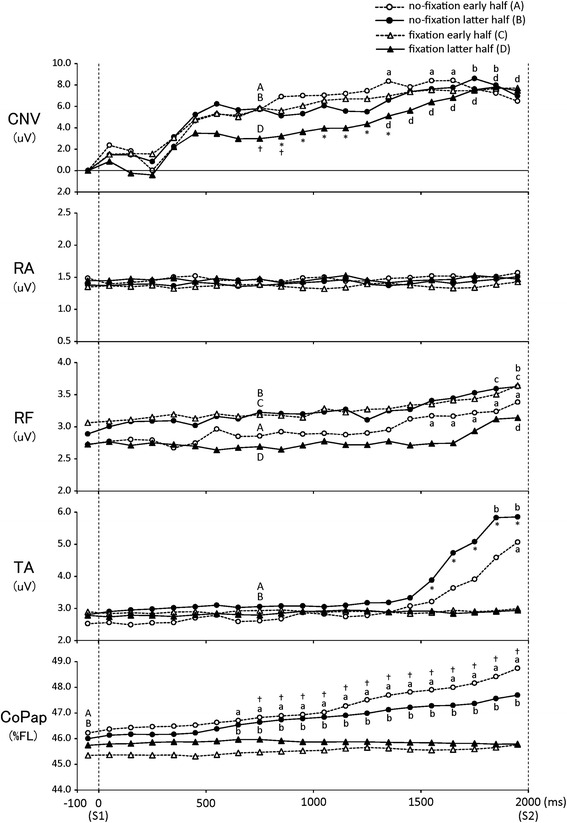
Mean amplitude for every 100-ms period of CNV, EMG, and CoPap between S1 and S2. *Significant difference between early- and latter-half sets (*p* < 0.05). †Significant difference between fixation conditions (*p* < 0.05). *Lowercase letters* indicate a significant increase compared with corresponding *capital letters* (*p* < 0.05)

Late CNV peaked just before S2 and then changed positively in 12 of 15 subjects. In the other three subjects, the negative potential was not clearly increased, and the following analysis between S1 and S2 was thus performed for the 12 subjects. Mean amplitudes for every 100 ms between S1 and S2 of CNV, EMG, and CoPap are shown in Fig. 5. CNV mean amplitude in the following periods was significantly larger than the 700–800 ms period: from 1300 to 1700 ms in the early half and from 1700 to 1900 ms in the latter half under no-fixation; and from 1400 to 2000 ms in the latter half under fixation (*p* < 0.05). In the period from 800 to 1500 ms, CNV mean amplitude was significantly smaller in the latter half than in the early half (*p* < 0.05). In the period from 700 to 900 ms, CNV mean amplitude was significantly smaller under fixation than under no-fixation (*p* < 0.05). No significant differences were seen across all four sets in CNV peak amplitude (mean across all sets: 9.6 (4.1) V). CNV peak time was significantly later in the fixation condition than in the no-fixation condition (*p* < 0.001), regardless of set (means between sets in the no-fixation and fixation conditions: −280.5 (163.1) ms and −171.8 (148.4) ms, respectively).

In the S1–S2 period (Fig. 5), CoPap mean position gradually deviated forward until S2 under the no-fixation condition. A significant forward displacement relative to the mean CoPap position before S1 was found in all periods after 600 ms in both halves under the no-fixation condition (*t*_11_ > 2.3, *p* < 0.05). Mean CoPap positions in all periods after 700 ms were significantly more forward under no-fixation than under fixation, regardless of set (*p* < 0.05). Mean TA amplitude tended to increase gradually until S2 under the no-fixation condition. A significant increase relative to the 700–800 ms period was found in the 1900–2000 ms period for the early half and in the periods after 1800 ms for the latter half (*p* < 0.05). Mean TA amplitudes in the periods after 1600 ms were significantly larger in the latter half than in the early half under the no-fixation condition. For RF, mean amplitudes tended to increase until S2 in all four sets. In particular, in the latter half under the fixation, the increase in RF mean amplitude tended to start just before S2. Under the no-fixation condition, a significant increase relative to the 700–800 ms period was found in the periods after 1500 ms for the early half and in the 1900–2000 ms period for the latter half. Under fixation, a significant increase relative to the 700–800 ms period was found in the periods after 1800 ms for the early half and in the 1900–2000 ms period for the latter half (*p* < 0.05). For RA, no tendency toward activation increase was observed until S2 and no significant differences were found between conditions and sets in every period.

**Fig. 6:**
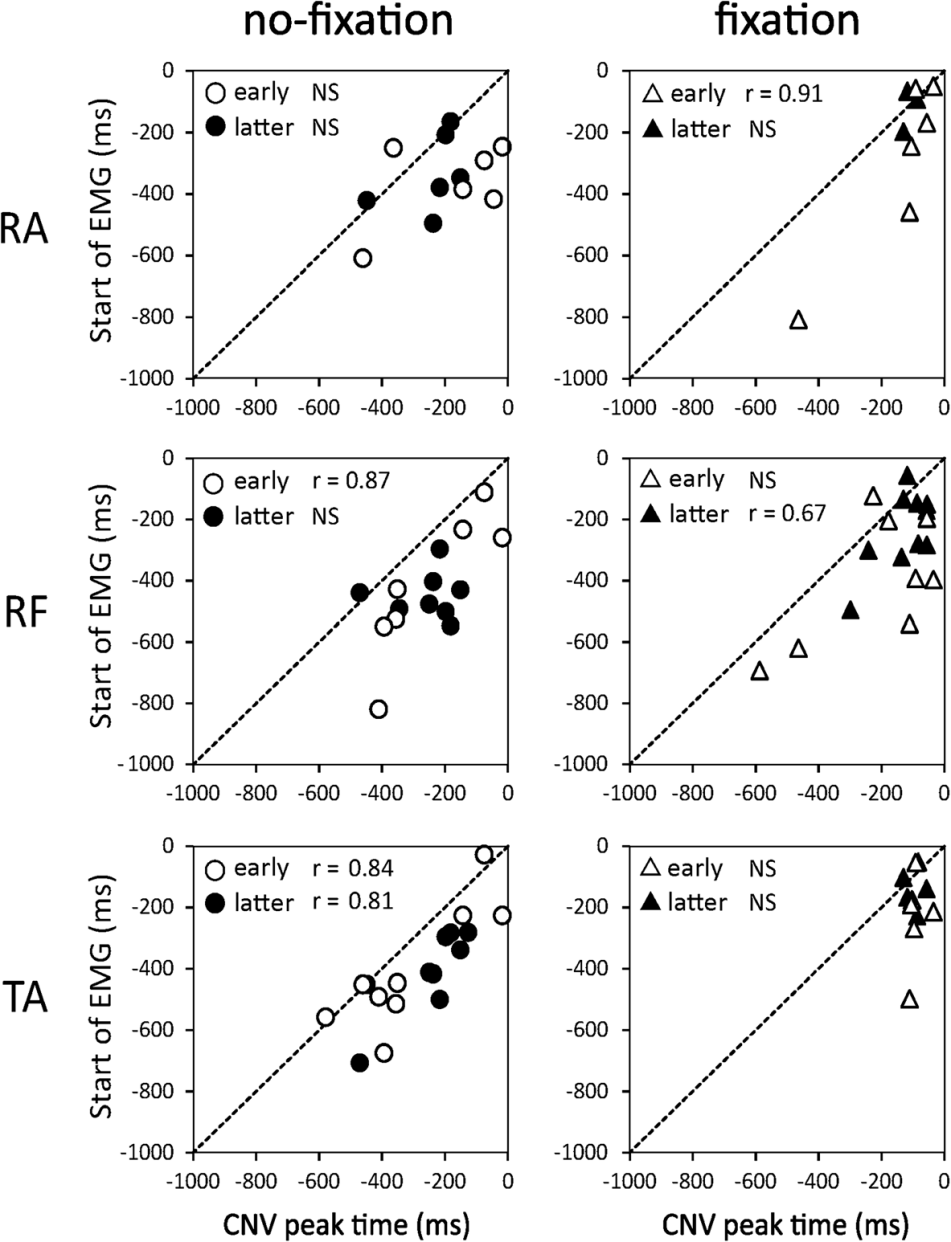
Relationships between CNV peak time and start time of EMG increase

Around the CNV peak point, the following EMG activations were found: a continuous increase from around CNV peak to S2 (no-fixation: early half 10; latter 6, fixation: early 10; latter 9) or a transient increase followed by forward displacement of CoPap (no-fixation: early 1, latter 5; fixation: early 3, latter 1). The EMG start time before S2 was similar across all 4 sets (mean −344.4 (195.2) ms). A slight increase in EMG slightly preceded the CNV peak (mean 118 ms), with no significant differences between them. Under no-fixation, a significant correlation between CNV peak time and EMG start time was found in TA (*r* = 0.84) and RF (0.87) in the early half, and in TA (0.81) in the latter half (all *p* < 0.05, Fig. 6). Under fixation, a significant correlation with CNV peak time was found in the RA (0.91) in the early half and RF (0.67) in the latter half (both *p* < 0.05).

## Discussion

Before adaptation in the no-fixation condition, CoPap was displaced to a position near EBL (Fig. 2), which is an index of the posterior limit of stability. During forward floor translation, the main joint for postural control is the ankle and TA activates as a focused postural muscle [3, 16]. In the present study, TA activated earliest and largest under the no-fixation condition (Fig. 4), suggesting that the focused postural muscle was the TA. CoPap displacement and TA activation decreased adaptively with trial repetition (Figs. 2 and 4).

Under the fixation condition, the peak latency of TA became longer and almost the same as the RF peak latency. The peak amplitude of TA decreased markedly, while that of RF increased significantly. The peak latency of RA became slightly shorter with fixation, but was still longer than those of RF or TA (Fig. 4). These results suggest that unification below the knee by ankle fixation would result in backward disturbance around the knee joint and a change in the main activation muscle for postural control to RF. The later latency of RA is attributable to the characteristics of viscoelastic body [4, 43]. Movement angle of the knee joint became slightly smaller (Fig. 3), which was attributed to the increase in RF activation. In addition, CoPap displacement, activation of TA and RF, and movement angles of ankle and hip joints decreased with postural adaptation under the fixation condition (Figs. 2, 3, and 4). These results suggest adaptation to the new postural control strategy. No adaptive changes in peak latency of all postural muscles were seen (Fig. 4), indicating that activation order of postural muscles did not change with postural adaptation. Thus, after adaptation with ankle fixation, the main activation muscle for postural control should still be RF, and activation would become more effective according to adaptation.

The CNV amplitude increased extremely until 800 ms from S1 before adaptation, regardless of ankle fixation, and increased slightly after that point. According to postural adaptation, the CNV amplitude tended to decrease during the period of 800–1500 ms after S1, but the peak amplitude did not show differences with ankle fixation or adaptation (Fig. 5). Thus, after adaptation, CNV was small until 1500 ms after S1 and then increased to the peak point, which was remarkable in the fixation condition. Late CNV reflects the motor preparation process and anticipatory attention directed to S2 [22, 23]. The amplitude just before S2 reportedly increases with a more difficult task or larger attention directed to S2 [20, 44]. In the present study, although the timing of peak attentional allocation became closer to S2 onset by ankle fixation, the amount of attentional allocation just before S2 would be equal among fixation conditions or adaptation states. Moreover, the timing of the start of allocation would become later with adaptation, particularly under fixation conditions. In previous studies using the S1–S2 task, in which S1 is a simple warning signal and S2 is a finger response or arm movement, no early CNV increase was found [21, 25]. On the other hand, in the S1–S2 task in which S2 was a transient floor translation, CNV increased early as in the present study, before adaptation [16]. However, with the considerable trial repetition (>90 trials), the negative potential of early CNV seemed to decrease [28, 45]. The decrease in early CNV amplitude after postural adaptation would indicate that according to postural adaptation, attention should not increase beforehand, especially in cases where attention was directed only to the knee joint because of ankle fixation.

In the S1–S2 interval, without ankle fixation, CoPap shifted early to a forward position and postural muscles activated relatively early for RF and just before S2 for TA. With ankle fixation, no forward shifts in CoPap or increases in TA were found, but RF activation increased toward S2 (Fig. 5). CoPap shift would represent the control of standing position to moderate postural disturbance [20]. The large background activity of postural muscles would be related to the increase in stiffness against the disturbance, leading to the above-mentioned early burst activation to S2. The inclination of the whole body pivoting at the ankles is a postural movement pattern to effectively translate the center of gravity [46]. Due to the immobility of the ankle joint under fixation, a postural strategy with increased postural muscle stiffness, but without a shift in standing position, would be selected, and the main activation muscle should be RF. Furthermore, after adaptation with ankle fixation, RF background activity increased rapidly from 400 ms before S2 (Fig. 5). This changing pattern of RF from before to after adaptation was similar to that of CNV. These results suggest that immediately after the ankle fixation, attention and/or muscle activity to the postural perturbation would anticipatorily be heightened in relation to the changes of somatosensory information by fixation or short-term memory. Furthermore, according to postural adaptation under the fixation condition, even when the timing of onset for the increase in RF stiffness becomes just before S2, postural disturbance can be moderated, resulting in a later start in direction of attention to S2.

The late CNV peak just before S2 has been suggested to correspond to a peak in anticipatory attention and/or onset of attentional shift to objects other than S2, such as sensory information and output of motor commands [20, 24]. This peak time correlated positively with the start time for the increase in muscle activation before S2 after adaptation for TA under no-fixation and RF under fixation (Fig. 6). With adaptation, although attention would be focused on sensory information processing related to the TA in many subjects under no-fixation conditions, the target of attention would change to RF with fixation.

## Conclusion

With joint fixation of the ankle, as the main joint in the postural control, the main activation muscle changed from TA to RF during forward floor translation. The early component of CNV became smaller with adaptation, especially under fixation conditions, suggesting a decrease in the need for anticipatory attention from the early stage. Correspondingly, the timing of the increase in RF stiffness may be delayed. After adaptation, a significant correlation between the timing of CNV peak and increased muscle activation just before S2 was found in TA with no-fixation and in RF with fixation, suggesting that under ankle fixation, the target of attention would change to RF with postural adaptation.

## Abbreviations

ANOVA, analysis of variance; BF, biceps femoris; C7, vertebra prominens; CNV, contingent negative variation; CoPap, center of foot pressure in the anteroposterior direction; EBL, extreme backward leaning; EEG, electroencephalogram; EMG, electromyography; EOG, electrooculography; ES, erector spinae; FL, foot length; GcM, gastrocnemius; LED, light-emitting diode; QS, quiet standing; RA, rectus abdominis; RF, rectus femoris; S1, warning stimulus; S2, imperative stimulus; SD, standard deviation; Sol, soleus; TA, tibialis anterior
